# Surface charge density and induced currents by self-charging sliding drops†

**DOI:** 10.1039/d4sm00205a

**Published:** 2024-04-10

**Authors:** Pravash Bista, Aaron D. Ratschow, Amy Z. Stetten, Hans-Jürgen Butt, Stefan A.L. Weber

**Affiliations:** a Max Planck Institute for Polymer Research Ackermannweg 10 55128 Mainz Germany; b Institute for Nano- and Microfluidics, TU Darmstadt Peter-Grünberg-Str. 10 64289 Darmstadt Germany; c Institute for Photovoltaics, University of Stuttgart Pfaffenwaldring 47 70569 Stuttgart Germany Stefan.Weber@ipv.uni-stuttgart.de

## Abstract

Spontaneous charge separation in drops sliding over a hydrophobized insulator surface is a well-known phenomenon and lots of efforts have been made to utilize this effect for energy harvesting. For maximizing the efficiency of such devices, a comprehensive understanding of the dewetted surface charge would be required to quantitatively predict the electric current signals, in particular for drop sequences. Here, we use a method based on mirror charge detection to locally measure the surface charge density after drops move over a hydrophobic surface. For this purpose, we position a metal electrode beneath the hydrophobic substrate to measure the capacitive current induced by the moving drop. Furthermore, we investigate drop-induced charging on different dielectric surfaces together with the surface neutralization processes. The surface neutralizes over a characteristic time, which is influenced by the substrate and the surrounding environment. We present an analytical model that describes the slide electrification using measurable parameters such as the surface charge density and its neutralization time. Understanding the model parameters and refining them will enable a targeted optimization of the efficiency in solid–liquid charge separation.

## Abstract 2

Sliding drops induce a bipolar current in subsurface electrodes beneath a hydrophobic substrate. We show how it depends on substrate properties and how it can be optimized for energy harvesting.

## Graphical abstract

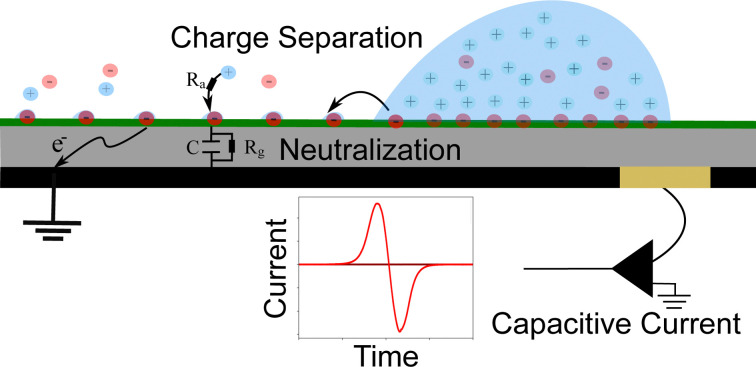

## 1 Introduction

Using water, one of the most abundant resources on earth, to harvest energy is not a new approach. From the kinetic energy of flowing water^1^ to the Kelvin dropper^2^ – all these processes use water as a resource. Recently, a new approach to generate electrical energy directly from slow flowing water has emerged. The method is based on solid–liquid contact charge separation on hydrophobic surfaces.^3^ Different groups are using sliding drops on top of these surfaces to build triboelectric nanogenerators (TENGs).^4–13^ Most TENGs involve a setup where drops sliding down the surface induce an electrical displacement in metal electrodes placed underneath the surface. This capacitive current caused by static charges in the moving drops is used to extract electric energy.^14–17^ Studies addressing the fundamental root of charge separation propose two different mechanisms for charge separation. While the transfer of ions from the solid–liquid to the solid–air interface is widely accepted,^16,18–21^ some argue with an additional electron transfer.^22–24^

Charge separation is a phenomenon observed at different solid–liquid interfaces.^23,25–30^ One fundamental aspect of solid–liquid contact is the electric double layer (EDL), which forms through the adsorption of ions from the liquid, protonation or deprotonation of surface groups, or the preferential dissolution of ions.^31^ When a sliding water drop leaves some of this charge on the dewetted surface after the drop, we call this effect slide electrification. During this process, the drop acquires an equal and opposite charge.^14,15,32,33^ Although some surfaces can acquire positive charges,^34^ most hydrophobic surfaces charge negatively while the sliding drops acquire positive charges. A possible mechanism of charge separation is that a certain fraction (*α*) of the surface charge within the EDL remains on the surface as the receding contact line moves.^14,16^ However, a direct measurement of the charge separation at the receding contact line has not been reported, so far.

The methods presented until now have quantified the drop charge and assumed that the opposite charge is left on the surface behind the drop. However, the quantification of the retained charge by the surface and surface charge neutralization are still missing. Here, we use a method inspired by TENGs to directly measure the solid–liquid charge separation as the contact line moves over the surface. Moreover, by comparing the surface charge left by grounded drops with the surface charge density in EDL, we can estimate how much of the surface charge from the EDL is left behind on the solid surface and how fast the charge is neutralized. Both of these quantities—the charge left on the surface and the surface discharge time—affect the measured capacitive current and thereby the harvested energy. Hence, understanding these processes and their dependencies on the specific materials and the environment can help to optimize energy harvesting.

## 2 Materials and procedures

### 2.1 Sample preparation

To prepare hydrophobic surfaces, we coated float glass and quartz glass slides (70 × 20 × 1 mm^3^) by chemical vapor deposition with PFOTS (trichloro(1*H*,1*H*,2*H*,2*H*-perfluorooctyl)silane, Sigma-Aldrich Chemie GmbH, Eschenstrasse 5, Germany). Here, we first cleaned the glass slides with acetone and ethanol. After that, we treated them in an oxygen plasma cleaner for 10 minutes at 300 W power (Diener Electronics Plasma surface: Femto BLS, Ebgausen, Germany), which should have removed all the organic compounds from the surface. Then, we placed the slides and a 1 mL vial of PFOTS with a magnetic stirbar in a vacuum desiccator. A pump evacuated the desiccator to a pressure of 100 mbar, which vaporized the PFOTS. Then the chamber was left on the stir plate for 30 minutes to complete the silanization process. After that, the surfaces were cleaned with ethanol and deionized water (DI) to remove unreacted PFOTS from the surface and left in a storage box for a few days prior to the experiments. The hydrophobic surfaces had advancing and receding contact angles of (120 ± 3)° and (87 ± 6)°, respectively, which were measured with a goniometer (Dataphysics, Germany).

### 2.2 Experimental procedure

**Fig. 1:**
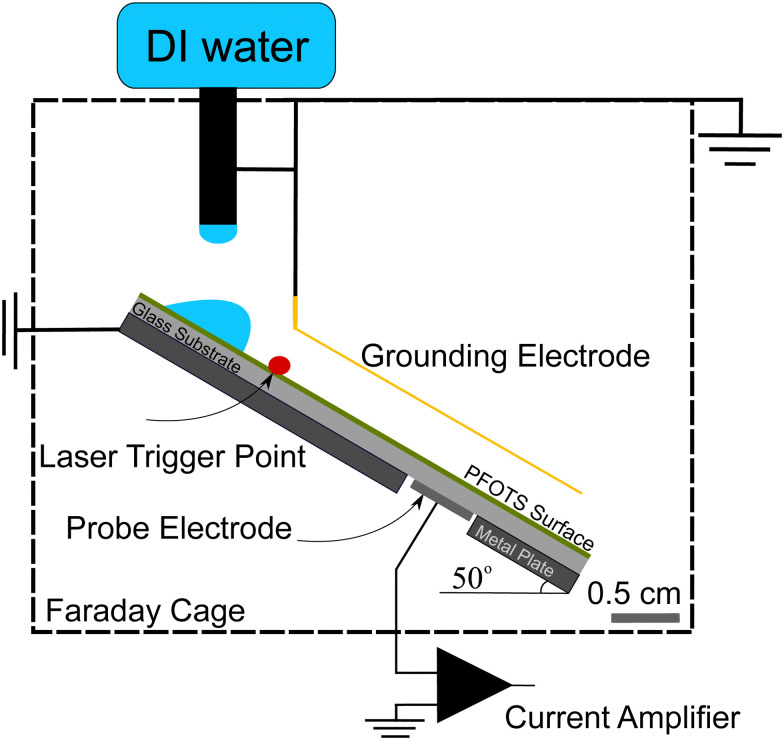
Schematic of the experimental setup. The whole setup is inside a Faraday cage and grounded. The setup is supplied with DI water (pH ≈ 5.5) using a pump. A drop of volume 45 μL falls from the height of around 0.5 cm and slides down the tilted plate. The sliding drop is then grounded using Au coated metal wire of 0.4 mm thickness. The capacitive current is measured using a current amplifier as the drop slides over the hydrophobic surface above the probe electrode. We use a National Instruments data acquisition board to record the current signal.

For the experiments (Fig. 1), we placed the samples on an inclined, grounded metal plate with a tilt angle of 50°. A circular open window was drilled into the metal plate to place a silver paint (few micrometers thickness) or copper (0.5 mm thickness) probe electrode of diameter 5 mm. We did not observe any difference in the measurement when using different probe electrodes. When a drop moved over the solid surface and acquired charge (Fig. 1), an opposite charge was left behind on the surface. We used the principle of a parallel plate capacitor to measure the capacitive current (*I*_c_) induced by this surface charge. Given that the plate length is *l* = 5 mm, which is five times larger than the distance between the plates (*d* = 1 mm), we assume a locally homogeneous electric field distribution and a low stray field within the capacitor as a first order approximation. Here, the sub-surface electrode acts as the bottom plate, while the sliding drop acts as the top plate of the capacitor. The induced capacitative current is given by 
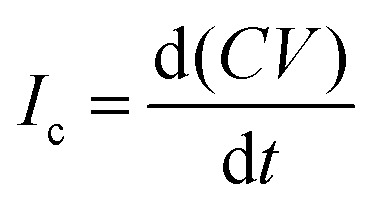
, where *V* is the voltage between the drop and ground, *t* is time, and *C* is the instantaneous capacitance of the drop-sub-surface electrode system. We used the simple sliding capacitor equations to model the induced capacitive current, as presented in a later section. By integrating the capacitive current traces, *I*_c_(*t*), we obtain the charge, 
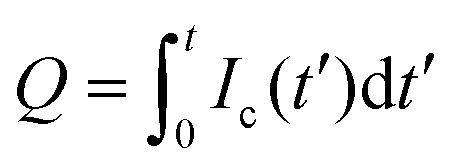
. We divide it by the electrode base area (2 × 10^−5^ m^2^) to get the surface charge density due to the sliding drop.

To ensure that the sample surface was neutral at the beginning of each experiment, we neutralized any previous surface charge using an ionizing air blower (Simco-Ion Aerostat PC Ionizing air blower) for 5 minutes before starting the experiment. The experiments were done at a temperature of 22 ± 1° and humidity of 30–50% and the tilted plate and the electrodes were all enclosed in a Faraday cage. A peristaltic pump (Gilson Minipuls 3, Wisconsin, USA) was used to pump DI water (Sartorius Arium Pro VF, 18.2 MΩ resistivity, Germany) into a grounded metallic syringe (diameter 2 mm, drop volume *V* = 45 μL). The drops fell from a height of (0.5 ± 0.2) cm and slid for (1 ± 0.2) cm to a first grounding electrode that defined the beginning of the sliding distance. To start the data acquisition, we used a laser trigger system positioned 20 ± 5 mm before the probe electrode. From this point, the capacitive current from the subsurface probe electrode beneath the substrate was recorded. Here, we used a sub femtoampere current amplifier (rise time: 0.8 ms, FEMTO DDPCA-300, Berlin, Germany) to record the current signal *via* a National Instrument data acquisition board (NI USB-6366 x-Series). Examples of such measurements are shown in the videos provided with ESI.†

We conducted two types of experiments: (i) to directly measure the surface charge left on hydrophobized glass, we placed Au-coated metal wire of thickness 0.4 mm parallel to the sliding drop path to continuously discharge the sliding drop. We also tried tungsten wire of thickness 0.025 mm as the grounding electrode and did not observe any difference. From the capacitive current to the sub-surface electrode we calculated the deposited surface charge (Fig. 1). (ii) To investigate the neutralization process of the surface charge, we varied the time between drops (Δ*t*) in a sequence of grounded sliding drops and measured the effect on the surface charge density (Fig. 5).

## 3 Results and discussions

**Fig. 2:**
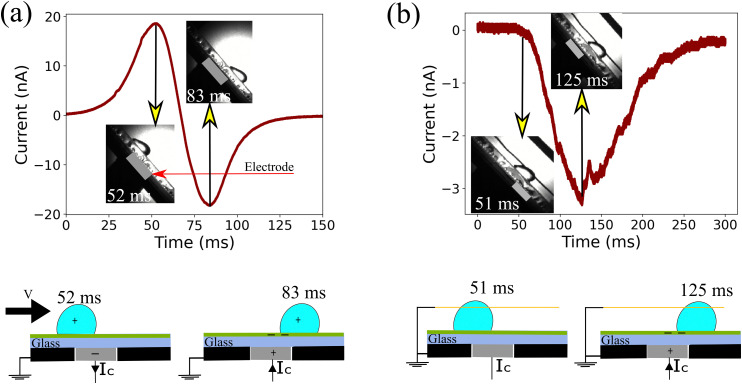
(a) Capacitive current and its corresponding drop position identified using high speed video for an ungrounded drop sliding down the solid surface. Capacitive current increases as the drop moves toward the probe electrode. It changes the polarity as the drop moves away from the probe electrode. (b) Capacitive current induced by the grounded drop sliding on top of the hydrophobic surface.

Most of the energy harvesting from the charge separation in sliding drops is done using the induced current in a metal electrode underneath the surface. The induced current in such devices is bipolar in nature.^24,35^ To understand this bipolar nature of the induced current, we first let an ungrounded drop run down the substrate (*i.e.* without a grounding wire). The passage of a drop with saturated drop charge (slide length *x* > 4 cm) over the probe electrode induced a bipolar current signal (Fig. 2a). To correlate the observed induced current with drop motion, we examined the drop position using a high speed camera (Photron FastCam Mini UX100, frame rate 1000, insets in Fig. 2). When the drop approached the electrode, we first observed a positive current as expected for a positively charged drop. The current peak at 52 ms happened when the drop position was a little more than half over the electrode, indicating that the majority of the drop charge is located at the rear of the drop. This off-centering is the result of the electrostatic interaction between the drop charge and the deposited surface charge after the drop.^17^ When the drop reached the center of the electrode, the current quickly flipped to negative, reaching a negative peak at 83 ms. The drop position with respect to the electrode edge at the negative current peak was again slightly off-centered. Our conclusion that the bipolar signal is connected to the image charges induced by the drop charge is further supported by experiments at different drop sliding lengths (ESI,† S2). At shorter slide length, the drops have not reached their maximum charge yet, and therefore, the induced current is weaker (Fig. S2, ESI†). For the saturated drop charge at a longer slide length, the induced current is larger.

To clarify the role of the static drop charge, we repeated the experiments with a grounded drop on a neutral surface (Fig. 2b). Here, we only observed a negative current peak. The absence of a positive peak is a clear proof that the working mechanism of capacitive TENGs is linked to the drop charge caused by the slide electrification. Nevertheless, as the drop moved over and away from the electrode, we observed a negative peak at 125 ms (Fig. 2b). We attribute this current to the negative charge left by the drop at the receding contact line. We only observed this negative current during the passage of the receding contact line over the electrode, clearly demonstrating that the charge separation occurs at the receding contact line. To study this surface charge transfer in more detail, the following discussion will address grounded drops, where we can neglect the influence of static drop charges.

### 3.1 Charge separation at the receding contact line

To demonstrate that the observed capacitive current signals are the result of slide electrification caused by moving water drops, we will conduct a quantitative analysis of the signal. We aim to show that the induced current is caused by the surface charge density left as the drop dewets the dry surface. Because of charge conservation and the insulating nature of both the substrate and the surrounding air, any change in total drop charge is the result of surface charges leaving and entering the drop. We recently proposed a model describing the charge separation based on the assumption that the charge density left on the surface, *σ*_out_, is a fraction, *α*, of the charge density in EDL, *σ*_EDL_:^16,33^

1 *σ*_out_ = *ασ*_EDL_.

To quantify the surface charge density, *σ*_out_, left by the grounded sliding drop on a neutral glass surface, we integrated the capacitive current (*I*_c_) induced by the sliding drop. By normalizing the charge by the electrode area (2 × 10^−5^ m^2^), we obtained a surface charge density of *σ*_out_ = −28 ± 2: μC m^−2^. We compared this value to the expected equilibrium charge density in the EDL with *σ*_out_ using the Grahame equation,^31^ which relates the zeta potential and the charge density2
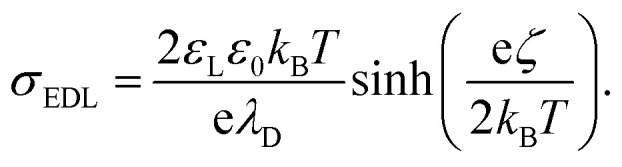


Here, *ε*_L_ and *ε*_0_ represent the dielectric constant of the liquid and vacuum, respectively. For this comparison, we used the zeta potential *ζ* = −36 mV at pH ≈ 5.5, as reported by Vogel *et al.*^36^ Using this *ζ*-potential and a typical Debye length of *λ*_D_ = 300–500 nm of distilled water in eqn (2), we estimated the surface charge density to be around −140 to −90 μC m^−2^. The comparison shows that all such measurements yielded 20–50% of the total charge density from EDL is left behind on the PFOTS coated substrate. Hence, understanding this fraction, *α*, and increasing it could help to increase the drop/surface charge, hence the induced current.

**Fig. 3:**
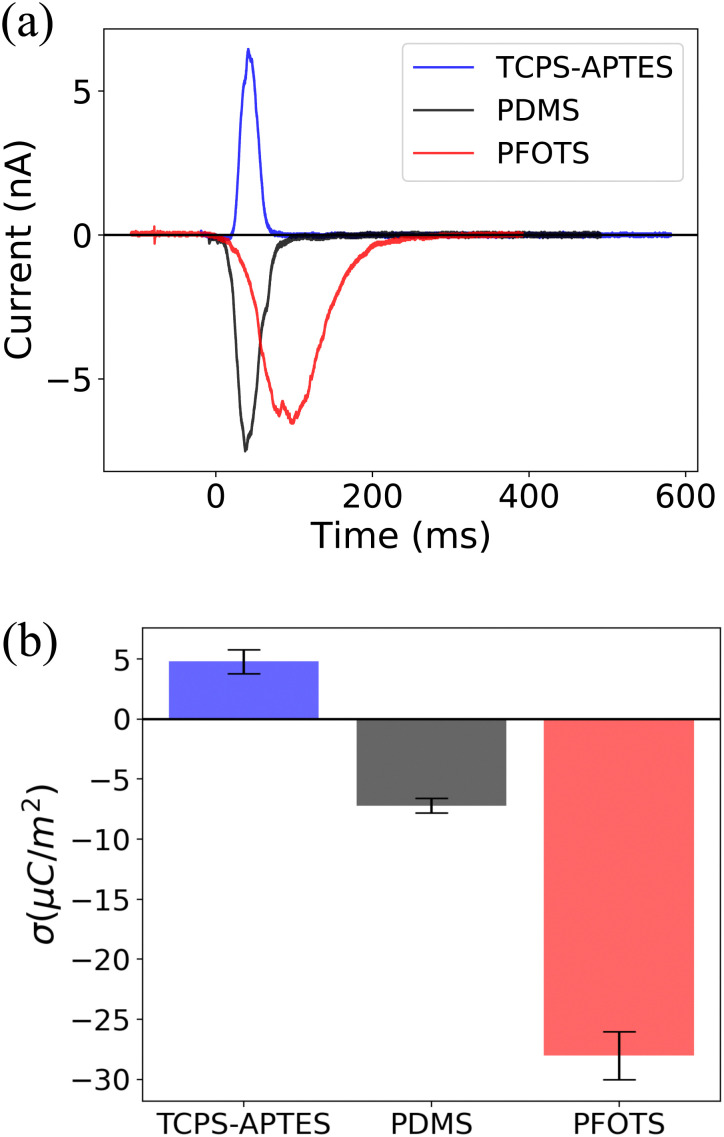
(a) Capacitive current induced by a grounded sliding drop on glass coated with different hydrophobic surface. (b) Surface charge density (*σ*) estimate integrating the current curves in (a). We observe that APTES has a positive surface charge density (positive current peak) whereas glass coated with PDMS and PFOTS has negative surface charge densities (negative peak).

We also measured the surface charge density of several other hydrophobized glass surfaces. Fig. 3 illustrates the surface charge densities of trichloro(propyl)silane (TCPS), (3-aminopropyl)triethoxysilane (APTES), polydimethylsiloxane (PDMS), and fluorocarbon (trichloro(1*H*,1*H*,2*H*,2*H*-perfluorooctyl)silane (PFOTS)) (details in previous work^34^). We observed a positive (*σ*_out_ = 5 ± 1: μC m^−2^) surface charge density for TCPS-APTES, potentially attributed to the presence of amino groups on the surface. Previous studies have indicated that the presence of APTES increases the surface charge density to a positive value, *e.g.* for nanoparticles coated with APTES,^37^ or for PDMS coated with APTES.^38^ The measured negative surface charge density for PFOTS and PDMS (*σ*_out_ = −7 ± 1: μC m^−2^) can be attributed to the presence of fluorine and hydrocarbon groups on the surface. These measurements confirm the correlation between the surface chemistry and slide electrification.

### 3.2 Surface discharge time

**Fig. 4:**
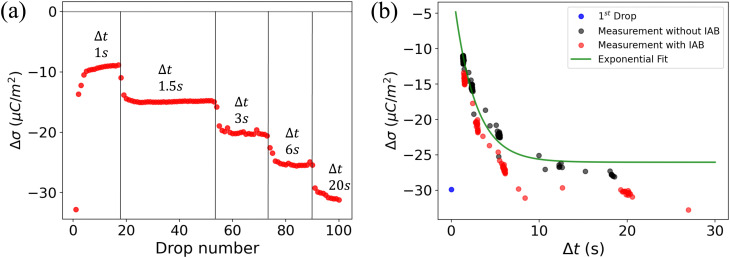
(a) Change in surface charge density, Δ*σ* (red dots) of PFOTS on float glass with increasing drop number and increasing drop interval. The drop interval was changed during the experiment by reducing the rotational rate in the peristaltic pump. (b) Measurement in float glass substrate. Steady-state Δ*σ* with increasing time between subsequent drops Δ*t*. The first drop, with *σ*_in_ = 0, is represented by a blue point. Measured data is fitted using eqn (5).

To understand the role of the substrate in surface discharge time and slide electrification, we allowed multiple grounded drops to slide down the surface. When a drop slides over a previously charged surface, the charges will be absorbed at the advancing contact line, whereas new surface charge is deposited at the receding contact line. We estimated the change in surface charge density due to the drop passage, Δ*σ*, by integrating the total capacitive current signal and dividing it by the electrode area. The measured values for Δ*σ* varied with drop number and the drop interval time, Δ*t* (Fig. 4a). The initial Δ*σ* was around −30 μC m^−2^ and quickly saturated at −10 μC m^−2^ within the first ten drops at a drop interval time of Δ*t* = 1 s. As we increased Δ*t* during the experiment, the steady-state Δ*σ* saturated at lower values, eventually reaching almost the value of the first drop at Δ*t* = 20 s.

The change in surface charge density as a drop slides at location *x* can be written as

3 Δ*σ*_*n*_ = [*σ*_in,*n*_(*x*) − *σ*_out,*n*_(*x*)]

where *σ*_in,*n*_ and *σ*_out,*n*_ are the surface charges entering the drop *via* the advancing contact line and leaving the drop at the receding contact line, respectively. They are equal to the surface charge densities of the solid–air interface directly before and behind the drop. As the surface is carefully neutralized prior to the experiments, there is no incoming surface charge density for the first drop; therefore, *σ*_in,1_ = 0.

**Fig. 5:**
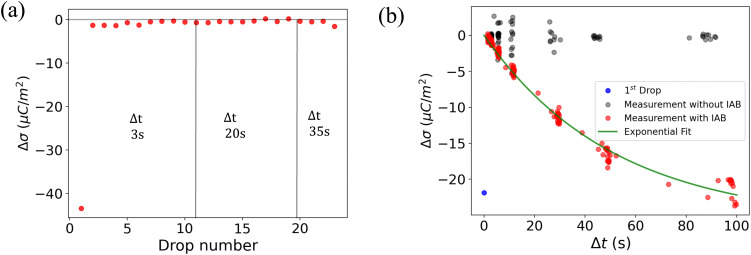
(a) Measured Δ*σ* (red dots) on PFOTS on quartz *versus* the drop number and increasing drop interval. (a) Measurement on quartz glass substrate. Steady-state Δ*σ* with increasing time between drop Δ*t*. The first drop (blue point) show the Δ*σ* after the first drop as red dots shows the Δ*σ* at different Δ*t* with ioning air blower (IAB) running in the background. Black dots show Δ*σ* with increasing Δ*t* but without IAB running in the background.

The dynamics in Δ*σ* with drop number and drop interval, as observed in Fig. 4a and 5a, can be explained when considering the surface charge caused by previous drops, denoted as *σ*_in_. Because of the finite time between successive drops, the surface loses some of the surface charges *via* discharge processes like conduction through the substrate^39–42^ or atmospheric ions caused by cosmic rays.^43^ With an exponential surface discharge time, *τ*, the incoming surface charge density can be estimated with4
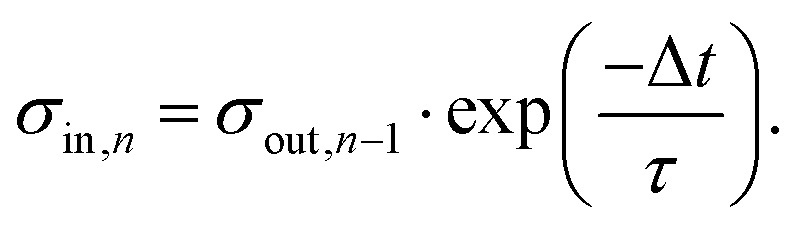


After a sufficiently large number of drops, the system reaches a steady-state, where *σ*_out_ and *σ*_in_ are stationary and where we can omit the drop number, *n*. Combining eqn (3) and (4), we obtain the change in surface charge density5
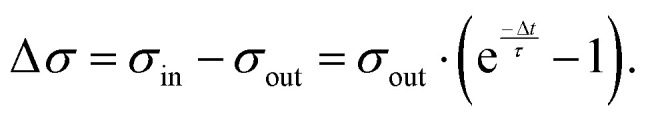


This equation describes the experimentally observed initial and steady-state Δ*σ* values, provided that we determine the surface discharge time, *τ*.

A way to measure the surface discharge time is to measure Δ*σ* with increasing time between drops, Δ*t*, as shown in Fig. 4a and b. As we increased the time between drops, the surface had more time to neutralize and the magnitude Δ*σ* increased, finally saturating at around −30 μC m^−2^, close to the value of the first drop. When the time between drops was too short (Δ*t* < *τ*), the steady-state Δ*σ* was around −10 μC m^−2^. For Δ*t* ≪ *τ*, Δ*σ* was close to zero. This was also observed in steady-state Δ*σ* in Fig. 5b. Given enough time between drops (Δ*t* ≫ *τ*), then Δ*σ* was equal to −*σ*_out_. Here, we estimated the surface discharge time *τ* = 2.4 ± 0.2 s for PFOTS coated glass substrate by fitting the data in Fig. 4b (black dots) with the eqn (5). We estimated the bulk decay current using *τ* in equation *σ*·*A*/*τ* to be 0.2 nA, which is lower than the sensitivity of the setup.

Measuring Δ*σ* on PFOTS-coated 1 mm thick quartz slides revealed a different behavior, as depicted in Fig. 5a and b. In this case, the surface charge density for the first drop was similar to that of the float glass. However, with increasing drop number and even at larger drop interval time, Δ*t*, the value for Δ*σ* remained close to zero (Fig. 4a and b). This result indicates that the surface becomes fully charged and is unable to discharge significantly in the time scale between drops. In fact, compared to float glass (soda lime glass), quartz has a factor 100 higher bulk resistivity.^44^

This observation clearly shows that under normal atmospheric conditions the substrate conductivity is the dominating factor for the surface discharge time (*τ*). Here, the surface discharge time could be understood as a capacitor discharge time, determined by the substrate's capacitance *C* and resistance *R*. To verify this argument, we measured the resistivity and capacitance of both float glass and quartz substrates. We applied an external voltage of 200 V to a stationary drop for 10 s and measured the capacitive current from beneath the substrate (details in ESI,† S3). The measurements on float glass revealed a resistance of *R* ≈ 2.5 × 10^12^ Ohm. Using a capacitance *C* ≈ 1.2 × 10^−12^ F (calculated as *C* = *ε*_0_*ε*_r_*A*/*d*), we estimated the discharge time to be 3 s, which closely aligns with the value obtained from the fit in Fig. 4b.

Interestingly, performing the same estimation in quartz using *R* ≈ 10^14^ Ω and *C* ≈ 0.7 × 10^−12^ F reveals a discharge time of hundreds of seconds. The difference in resistance can explain the variations observed in Δ*σ* between float and quartz in Fig. 4 and 5. This observation also emphasizes the significant role of the substrate in the surface discharging process. Using a low-resistivity substrate with short discharge times could be a promising approach to optimize the charge separation process in series of drops and could thus increase energy harvesting efficiency.

To further understand the influence of atmospheric ions in the discharge process, we repeated the above experiment with an ionizing air blower (IAB) turned on during the experiment. The IAB was positioned at a 45° angle to minimize the effect of air motion on the sliding drops. We measured a concentration of more than 300 000 ions per cm^3^ atmospheric ions directly at the substrate using an Ionometer (IM806v3, 66687 Wadern, Germany).

In Fig. 4b and 5b, the red dots show the measurements with the IAB. In the case of glass, using the IAB has little effect on the discharge process, supporting the conclusion that the direct discharge through the glass substrate is dominant. However, in the case of quartz, where the discharge through the substrate is suppressed, we now observed a considerable increase of Δ*σ* with increasing Δ*t*. Here, the discharge through atmospheric ions seems to play an important role. To determine the discharge time on the quartz substrate in the presence of the IAB, we fitted the data (red dots) with an exponential curve (Fig. 5b) and estimated the discharge time to be approximately 50 s.

These experiments clearly demonstrate the respective role of atmospheric ions and the substrate conductivity in the surface discharging process. Under normal atmospheric conditions (air ion density of 100–200 ions per cm^3^), the influence of the substrate is much greater than that of the air ions. It also highlights that quartz can effectively hold the charge for an extended period. Interestingly, this does not seem to be the case for float glass. The discharge time in float glass is only a few seconds, and atmospheric ions play a minimal role.

### 3.3 Numerical simulation of capacitive current

We further extended the model with charge density, as discussed above, and were able to simulate the capacitive current. We propose a simple sliding plate capacitor model to verify the working principle discussed earlier. The concept involves the bottom electrode acting as the static bottom plate, while the charged sliding drop acts as the top plate. We assume that the saturated drop is at a constant potential *U*_drop_ ≈ 1–4 kV.^33^ While the drop slides over the electrode, the effective capacitor area changes, resulting in a change in capacitance. Together with the voltage in the capacitor, this dynamic capacitance leads to a capacitive current in the probe electrode.

The induced current due to slide electrification can be modeled using four coupled equations describing a sliding of charged plates in a capacitor as follows:6
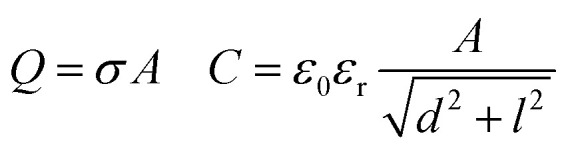
7
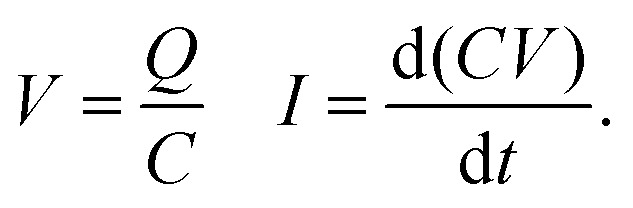


Here, *σ* is the charge density left on the surface, *A* is the area which changes while the drop is sliding over the bottom electrode, *d* is the dielectric substrate thickness, *l* is the horizontal distance between the sliding drop and the probe electrode (corresponding to the horizontal drop position), *V* is the voltage in the capacitor, and *t* is the drop sliding time which we calculated using the velocity (*t* = *l*/*v*).

First the starting physical parameters such as dielectric constant (*ε*_r_ ≈ 3.8 for quartz and 6 for glass), permeability (*ε*_0_), substrate thickness (*d* = 1 mm), and the width of the plate (*w* = 0.5 cm) were initialized in the simulation. Assuming a constant velocity of the drop, we calculated the time it takes for the drop to traverse the length of the probe electrode. Subsequently, we numerically solved eqn (6) and (7) for the sliding drop to calculate the change in *A*, *V*, and *C* over time and stored in an array. Using this array, we computed the resulting capacitive current and plotted it against time. The model is also able to simulate the induced current in surfaces like TCPS-APTES on glass, which have positive surface charge density, as shown in Fig. S4 in the ESI.†

**Fig. 6:**
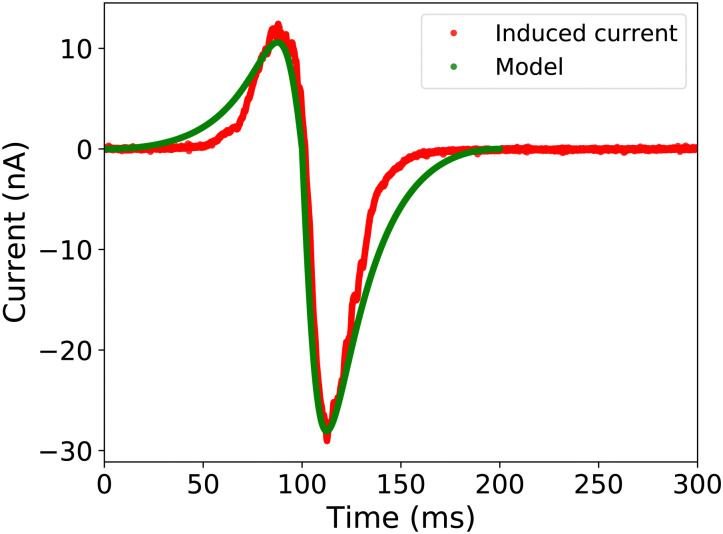
Induced capacitive current shows qualitative and quantitative agreement with the sliding plate capacitor model. We used *σ*_out_ = −28 μC m^−2^, *σ*_in_ = *σ*_out_ exp(−Δ*t*/3 s), and *v* = 0.15 m s^−1^ for the simulation.

We compared the simulated electrode current with the measurements, as illustrated in Fig. 6. For the simulation, we used a charge density of *σ*_out_ = −28 μC m^−2^ and a velocity of 0.15 m s^−1^, close to the experimentally observed values. Although the simulation uses a simplified rectangular drop shape, there is a good quantitative agreement between the model and the measurements. The slight deviation between the model and the measurements between 150 and 200 ms could be attributed to the simplified ellipsoidal drop shape and changing drop velocity due to the presence of the grounding wire.

## 4 Conclusion

Here, we investigated the charge transfer mechanism in water drops sliding down an insulating hydrophobic surface. Our approach is based on the principle of mirror charge detection in a sub-surface electrode, where we measure the capacitive current generated by the moving drop. By comparing the current traces generated by floating-potential and grounded drops and correlating the signals to the drop position, we confirm (i) that the working mechanism of droplet-based capacitive TENGs is linked to static drop charges and (ii) that the charge transfer during slide electrification takes place at the receding contact line. By comparing the generated charge density behind a drop with the zeta potential of the respective solid–water interface, we estimate the ratio of charge remaining on the surface to be around 20–50%. Our observations on multiple grounded drops sliding down the surface suggest that the ability of the substrate to retain the deposited surface charge is influenced by both the substrate conductivity and the environmental conditions. This neutralization process can be described by a characteristic discharge time. These insights not only enhance our understanding of slide electrification but also have the potential to be utilized in improving droplet-based nanogenerators by leveraging these effects.

P. B., S. A. L. W. proposed the work, P. B., A. D. R., A. Z. S. and S. A. L. W. proposed the measurement methods, P. B. prepared the substrates, conducted the experiments, and analyzed the data, A. D. R. derived the theoretical framework, S. A. L. W. and P. B. developed the model, P. B. carried out the simulations, P. B. and S. A. L. W. prepared the manuscript with input from all authors, H.-J. B. and S. A. L. W. supervised the work.

## Conflicts of interest

There are no conflicts to declare.

## Supplementary Material

SM-020-D4SM00205A-s001

SM-020-D4SM00205A-s002

SM-020-D4SM00205A-s003

SM-020-D4SM00205A-s004

SM-020-D4SM00205A-s005

SM-020-D4SM00205A-s006

SM-020-D4SM00205A-s007
