# Supplementary figures and images for: Surface charge density and induced currents by self-charging sliding drops
Source: Soft Matter. 2024 Apr 10;20(26):5045–52. doi: 10.1039/d4sm00205a (PMC11220910; doi:10.1039/d4sm00205a)

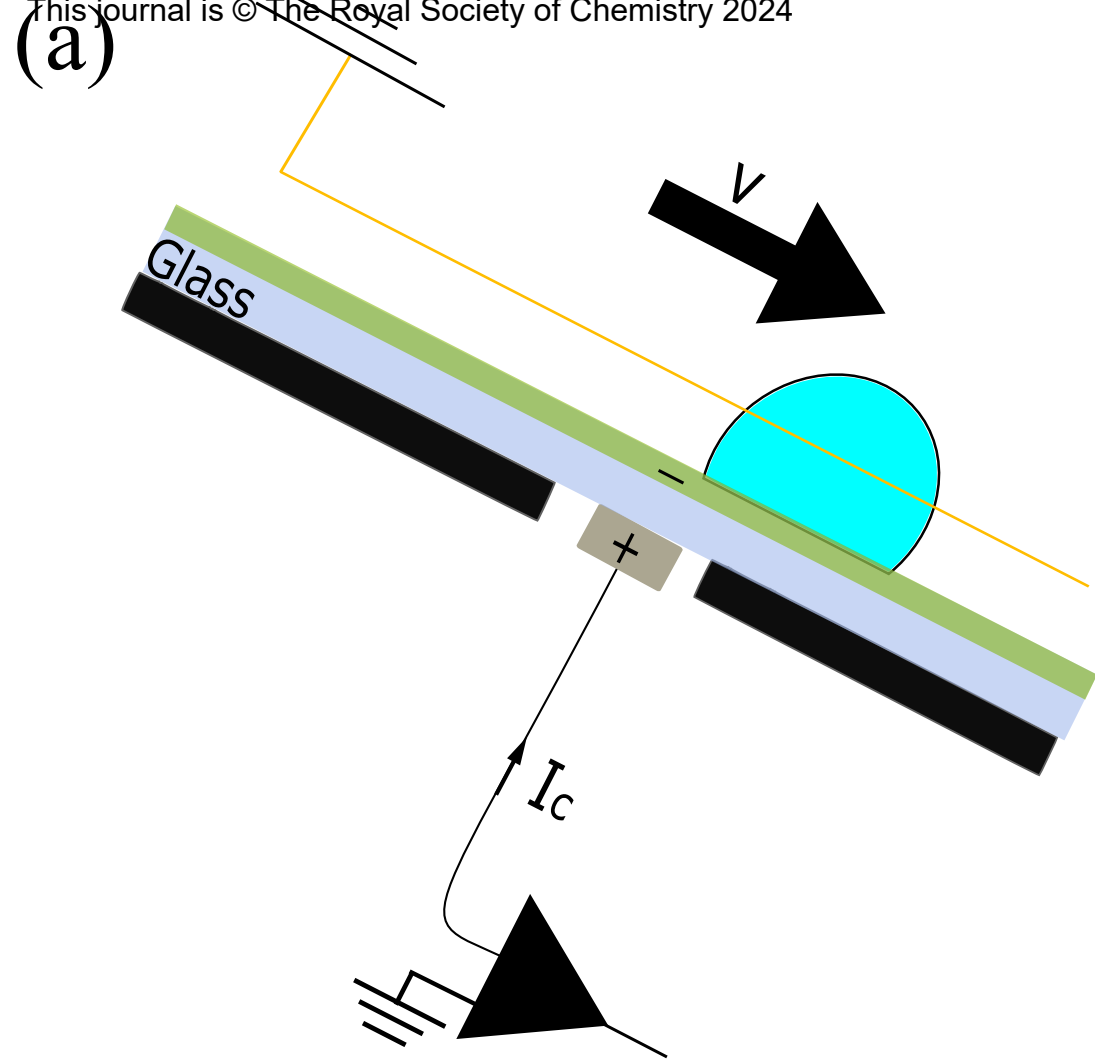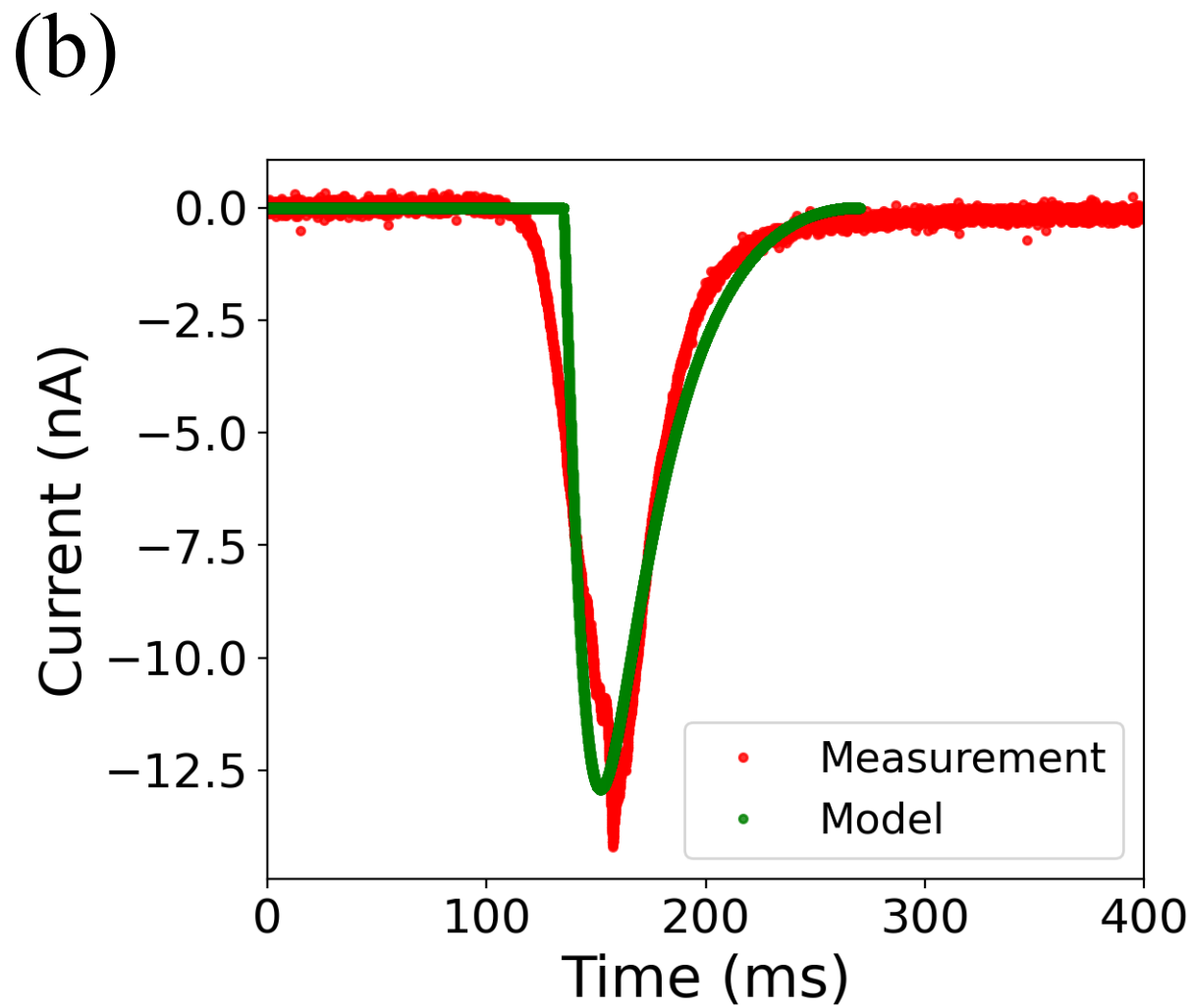

Supplement: SM-020-D4SM00205A-s003 [file SM-020-D4SM00205A-s003.pdf]

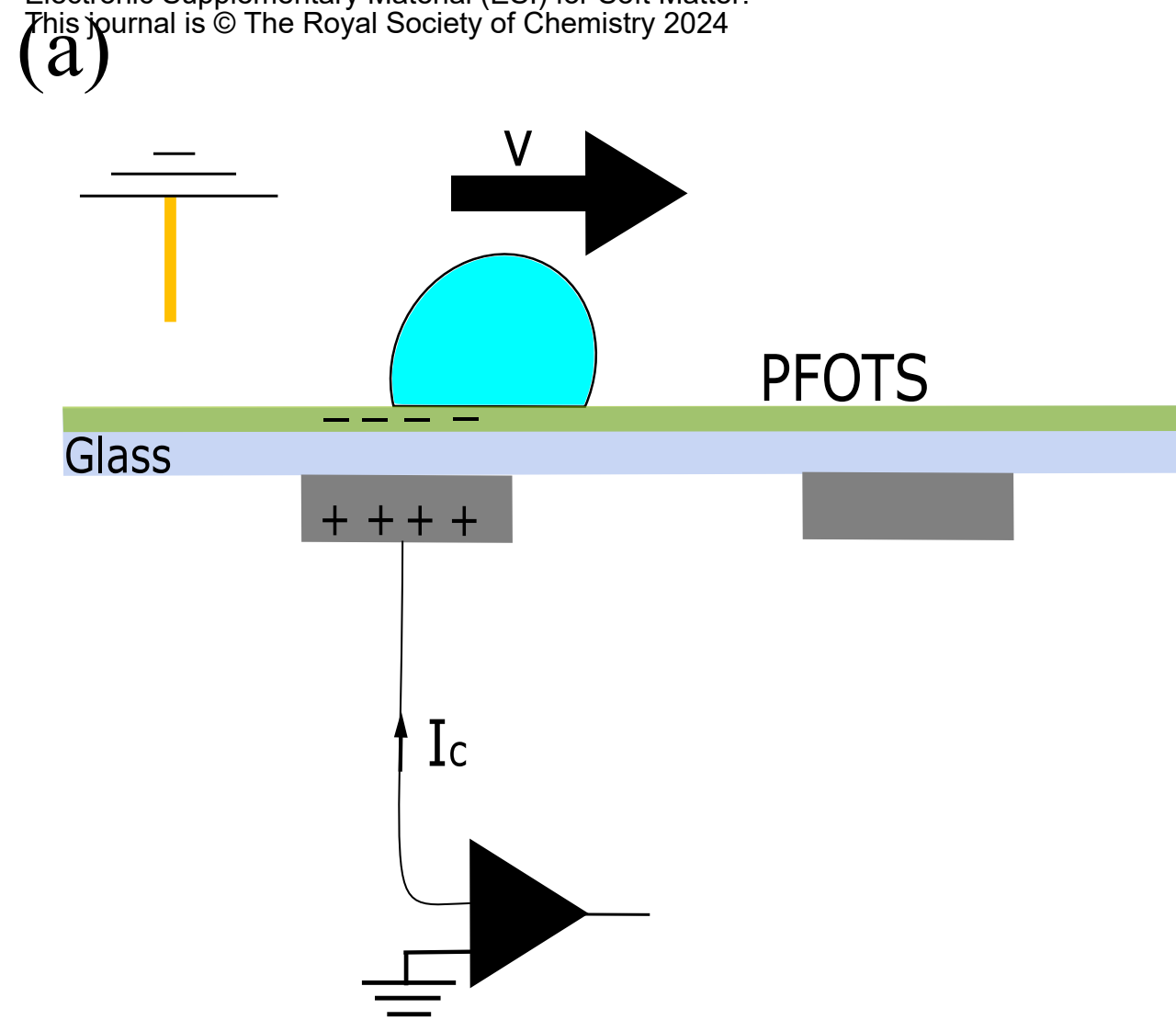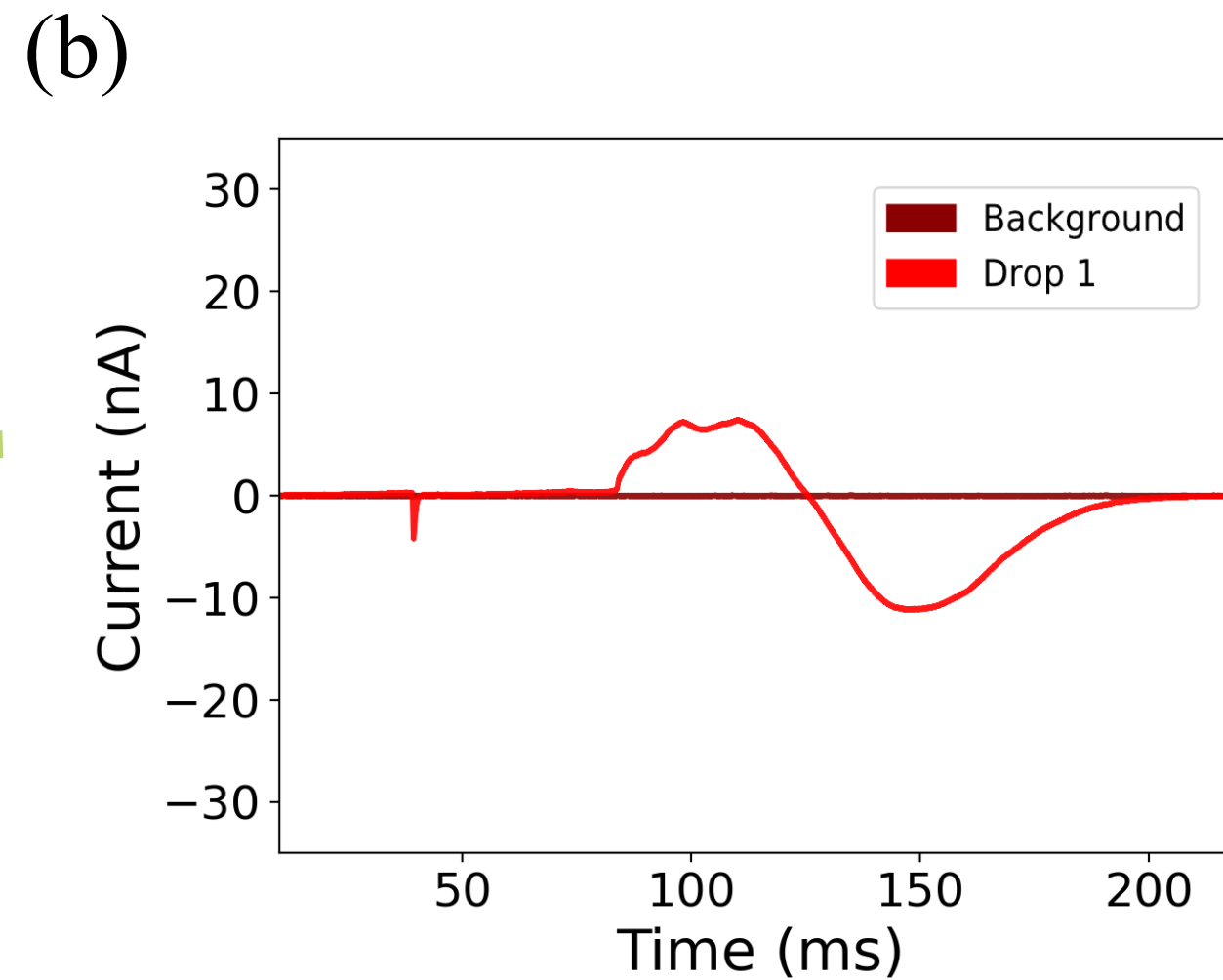

Slide length 1 cm

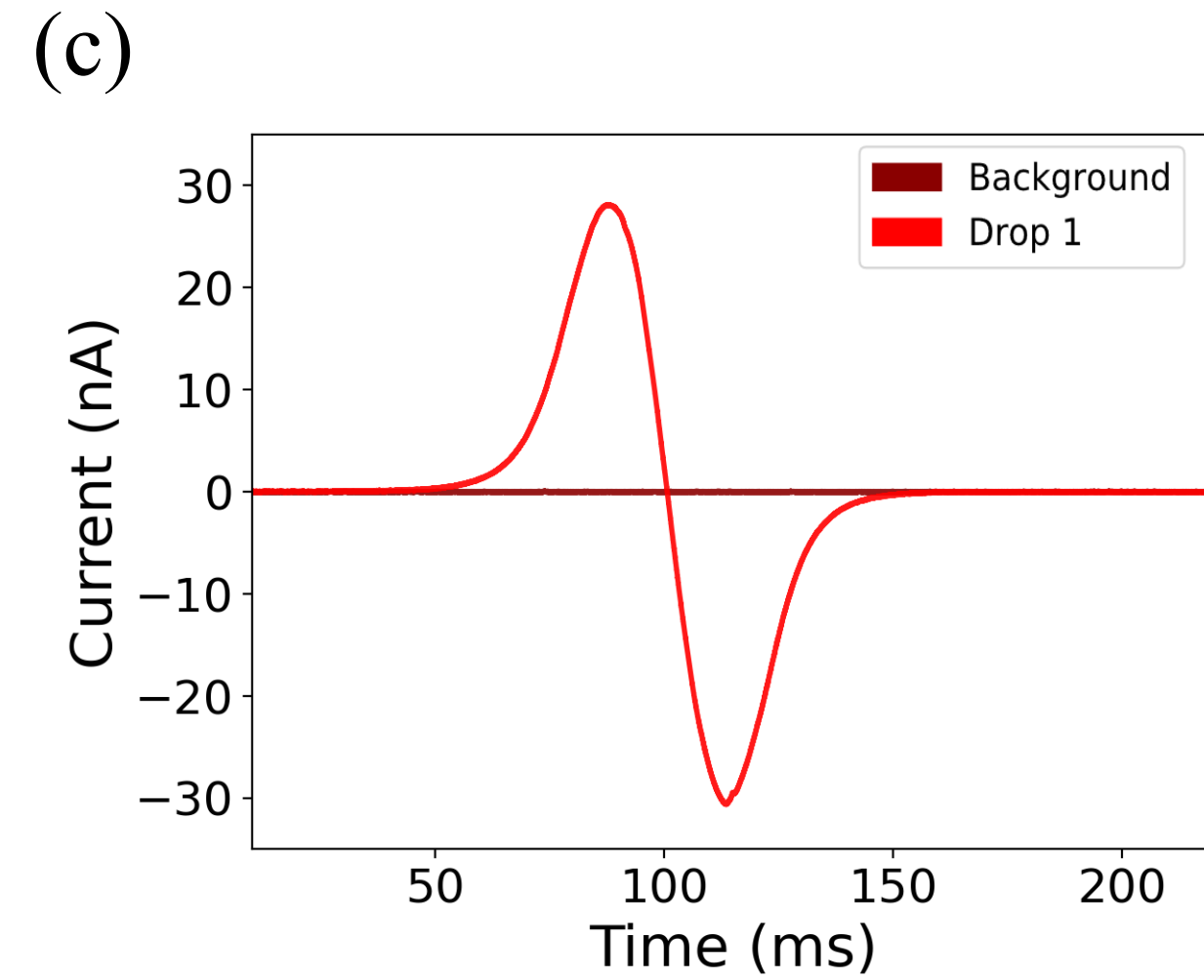

Slide length 5 cm

Supplement: SM-020-D4SM00205A-s004 [file SM-020-D4SM00205A-s004.pdf]

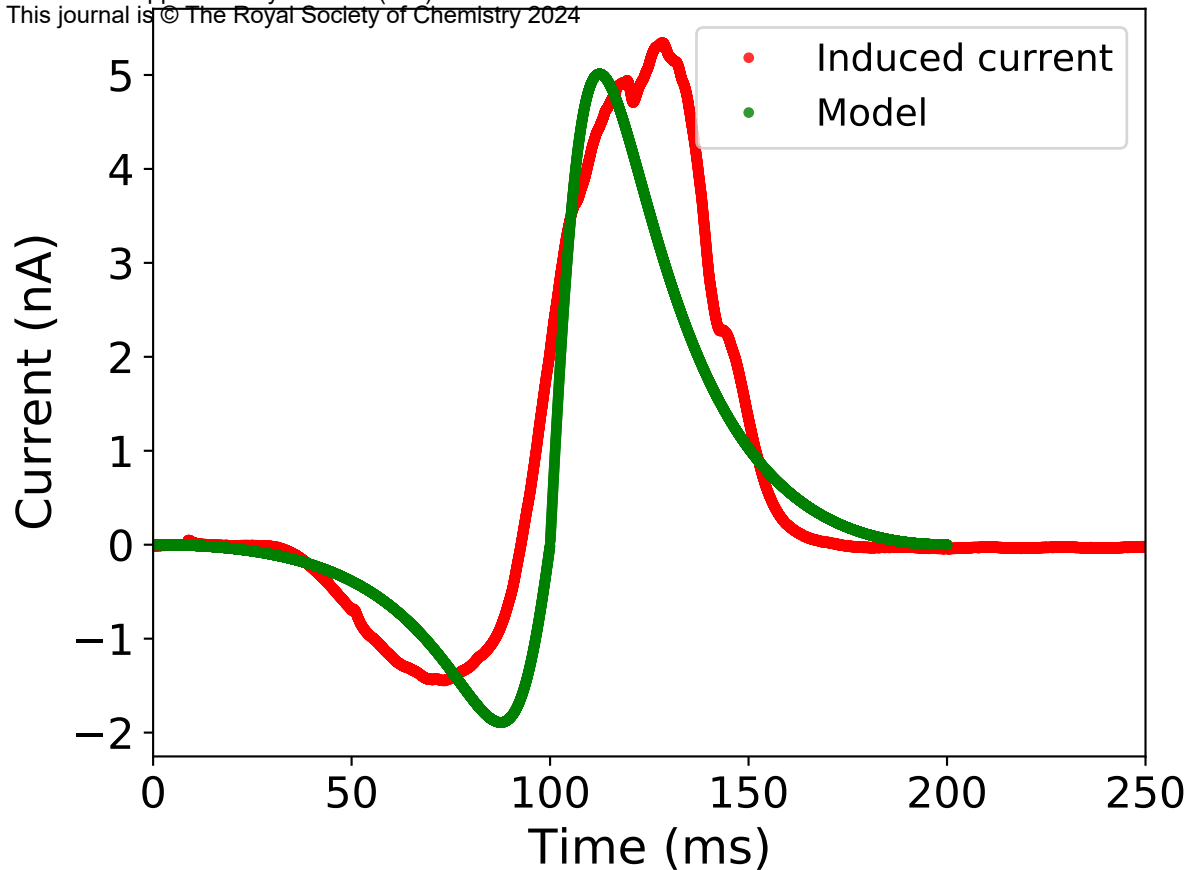

Supplement: SM-020-D4SM00205A-s005 [file SM-020-D4SM00205A-s005.pdf]

(a)

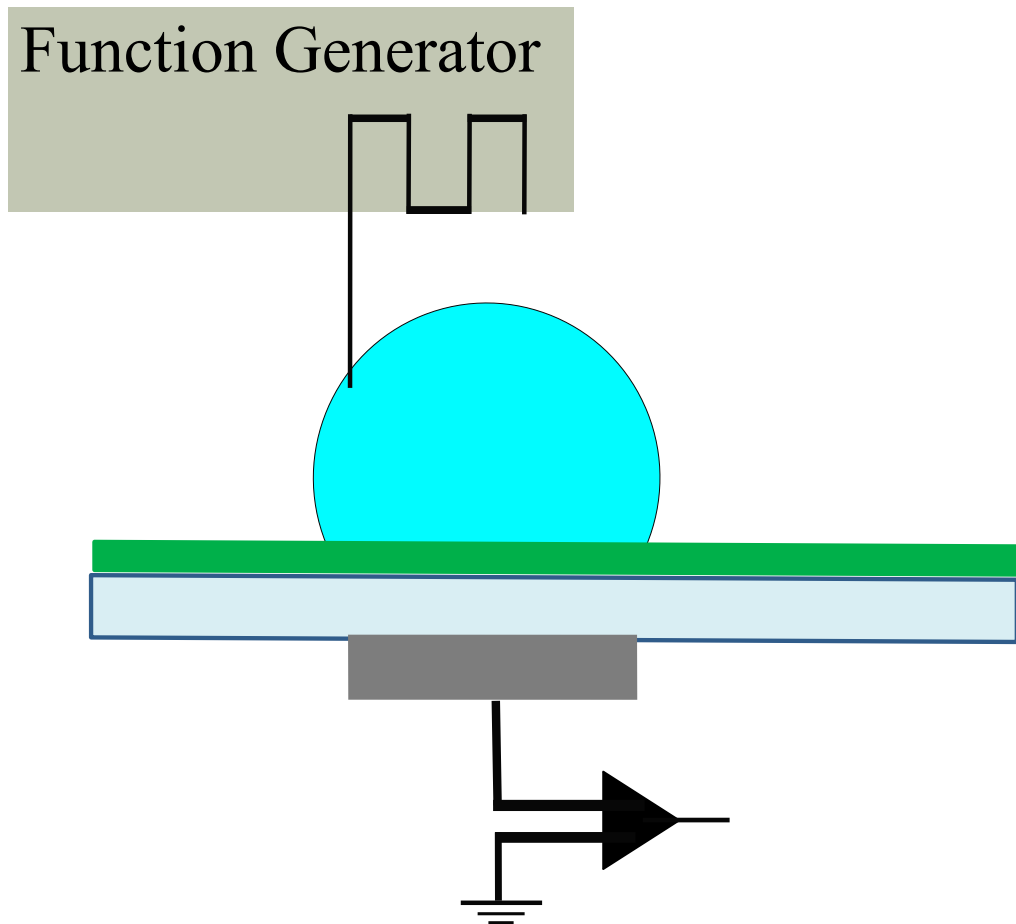

(b)

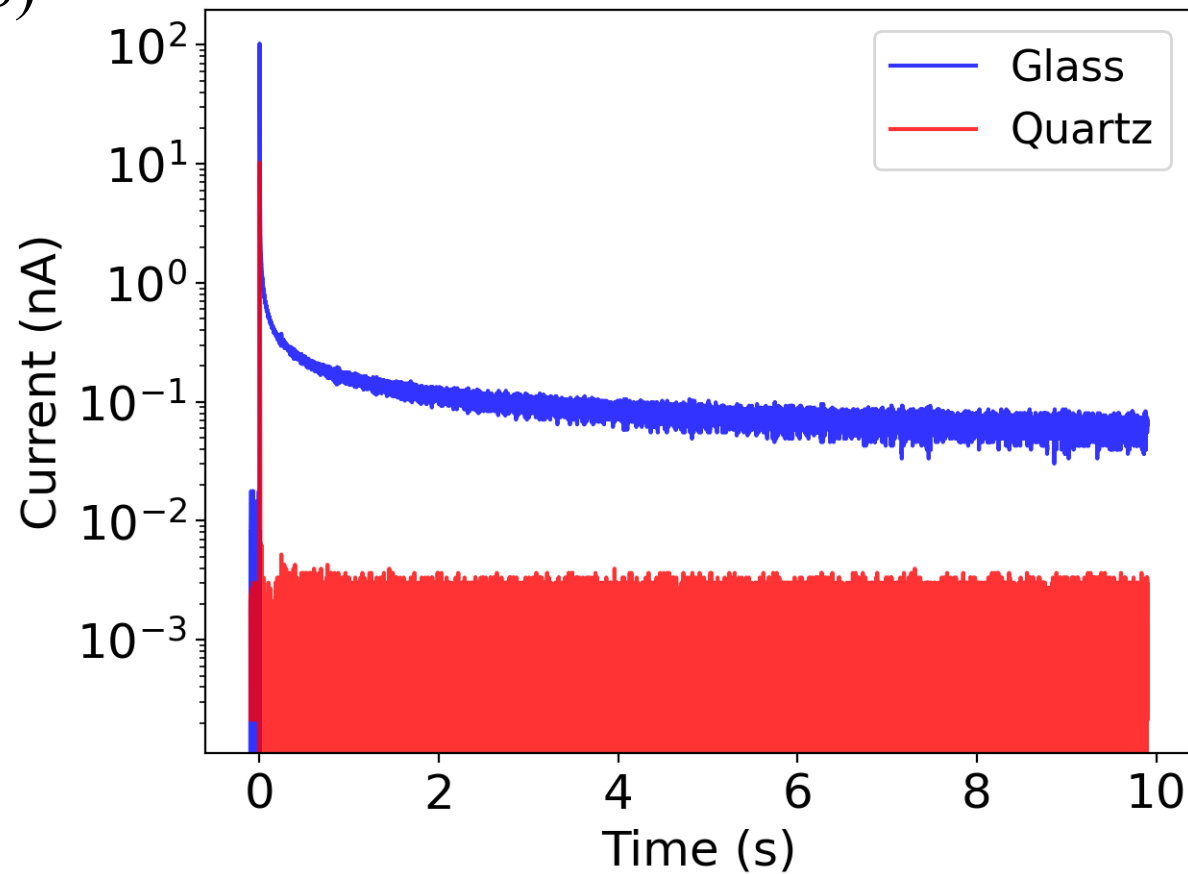

Supplement: SM-020-D4SM00205A-s006 [file SM-020-D4SM00205A-s006.pdf]
