# Supplementary material for: Surface charge density and induced currents by self-charging sliding drops
Source: Soft Matter. 2024 Apr 10;20(26):5045–52. doi: 10.1039/d4sm00205a (PMC11220910; doi:10.1039/d4sm00205a)
Supplement: SM-020-D4SM00205A-s007 [file SM-020-D4SM00205A-s007.pdf]

Electronic Supplementary Material (ESI) for Soft Matter.
This journal is © The Royal Society of Chemistry 2024

Surface Charge Density and Induced Currents by Self-Charging Sliding Drops

Pravash Bista[a], Aaron D. Ratschow[b], Amy Z. Stetten[a], Hans-Jürgen Butt[a], Stefan A.L. Weber[a,c]

[a] Max Planck Institute for Polymer Research, Ackermannweg 10, 55128 Mainz, Germany
[b] Institute for Nano- and Microfluidics, TU Darmstadt, Peter-Grünberg-Str. 10, 64289 Darmstadt, Germany
[c] Johannes Gutenberg University, Department of Physics, Staudingerweg 10, 55128 Mainz, Germany

Correspondence: webers@mpip-mainz.mpg.de

2024-04-03, 08:50:22

Contents

S1 Surface charge density on hydrophobized coated glass S2

S2 Capacitive current with increasing slide length and velocity S2

S3 Measuring conductivity of float and quartz glass S3

S4 Induced current in TCPS-APTES S3

S1 Surface charge density on hydrophobized coated glass

Figure S1a shows a setup for studying the surface charge density of a dielectric coated glass surface. In this setup, a grounded drop is made to slide down the surface with a certain velocity and the capacitive current I_c is measured, as shown in Figure S1b. We only observe the capacitive current as the drop slides away from the electrode. By integrating this current, we estimated the total charge generated by the sliding drop.

We further utilized the measured surface charge density and the change in area/capacitance with time to model the capacitive current, as shown in Fig S1b, which qualitatively matches the measurements. This simple model captures the parametric dependency of the capacitive current, relying on the velocity of the sliding drop, drop size, and the substrate's thickness. Consequently, to increase the capacitive current while maintaining the same charge amount, one must increase the velocity and drop size while decreasing the substrate thickness. This effect is also evident in our experiment: as the drop slides down the surface, both its velocity and the measured capacitive current signal increase (S2).

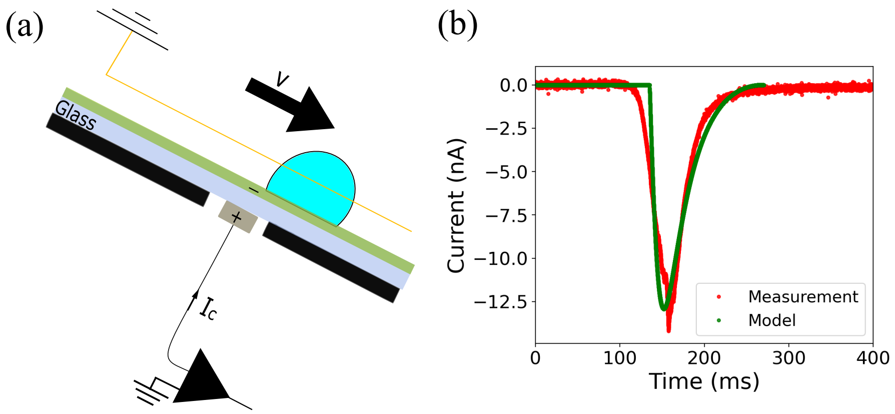

Figure S1: (a) Experimental setup to measure a capacitive current of sliding grounded drop. (b) The capacitive current of grounded drop sliding on top of the hydrophobic surface and the sliding capacitor model (green line) modelled using $\sigma = 28\frac{\mu C}{m^2}$ and velocity $v = 0.035\frac{m}{s}$

S2 Capacitive current with increasing slide length and velocity

An experimental setup for slide length study is illustrated in figure S2a. To measure the capacitive current with increasing slide length, we first neutralized the sliding drop using a ground electrode. Then, we let the drop slide down the surface and measured the capacitive current generated by the sliding drop. Figure S2 b and c displays the capacitive current measured at two different slide lengths. At 1 cm, the capacitive current from the incoming drop was less than that from the outgoing drop. However, at longer slide length of 5 cm, the capacitive current increased as the charged drop slid faster down the surface.

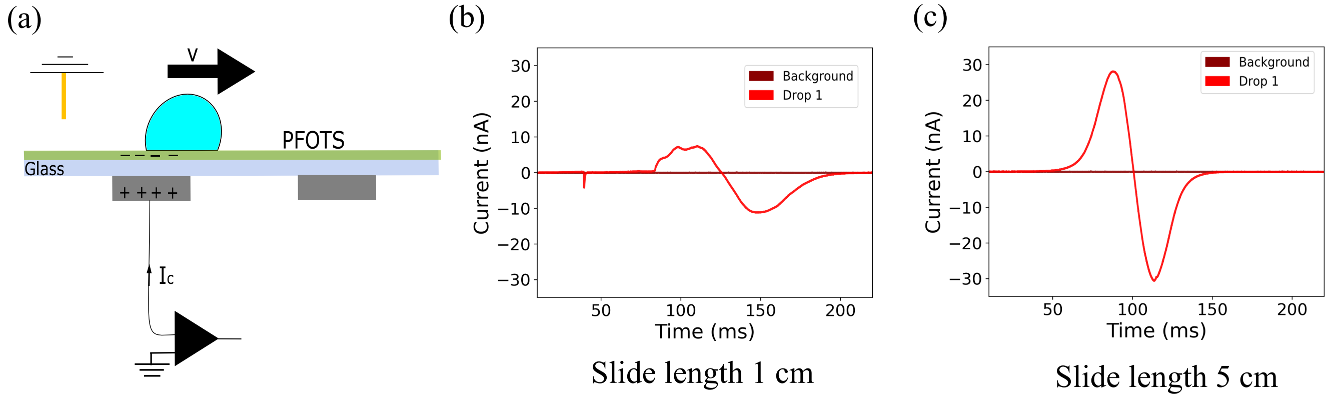

Figure S2: Capacitive current measure at different slide length as drop slide down the surface.

S3 Measuring conductivity of float and quartz glass

Figure S3a shows a setup for measuring the conductivity of float glass and quartz glass. In this setup, a plate capacitor system with a drop as the top plate is used. An external voltage of 200 V is applied for 10 seconds, and the capacitive current is measured. In Figure S3b, we observe that the saturation current value for glass is $\mathcal{O}(100)$ higher than that of quartz glass.

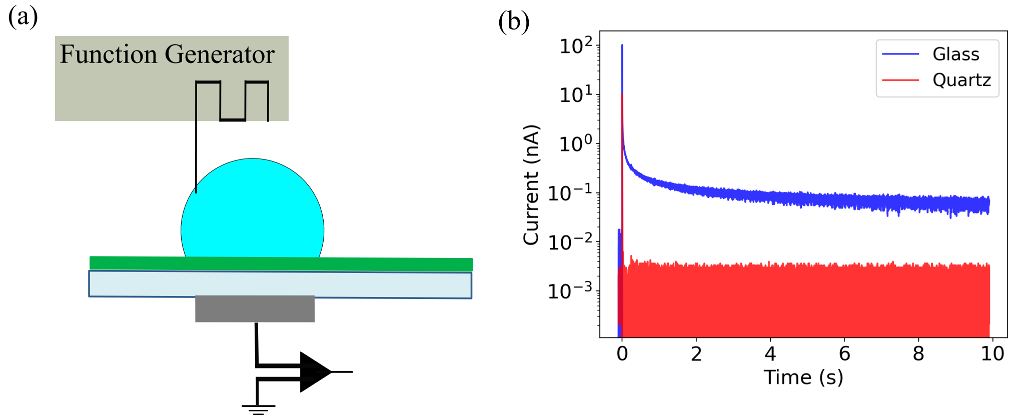

Figure S3: (a) Setup to measure capacitive current due to applied voltage. (a) Current for 10 s while applying external potential of 200 V

S4 Induced current in TCPS-APTES

We simulated the induced capacitive current on trichloro(propyl)silane (TCPS) and (3-aminopropyl)triethoxysilane (APTES) (TCPS-APTES) on glass as shown in Figure S4.The amine-doped positively charging surface in contact with water with a pH of approximately 5 was also reported in our previous work[1]. The model is capable of simulating both the positive and negative polarity of induced current.

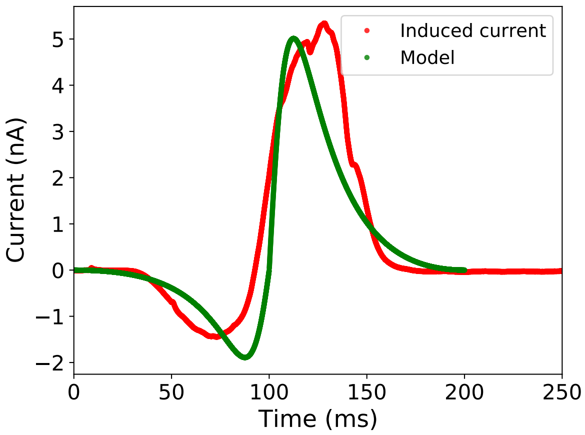

Figure S4: Induced capacitive current shows qualitative agreement with the sliding plate capacitor model. We used $\sigma_{out} = 5\ \mu C/m^2$, $\sigma_{in} = \sigma_{out} exp(-\Delta t/3)$, and $v = 0.15\ m/s$ for the simulation.

References

[1] W. S. Wong, P. Bista, X. Li, L. Veith, A. Sharifi-Aghili, S. A. Weber and H.-J. Butt, *Langmuir*, 2022, **38**, 6224–6230.
